# Factors associated with testing for HIV and hepatitis C among behaviorally vulnerable men in Germany: a cross-sectional analysis upon enrollment into an observational cohort

**DOI:** 10.1186/s12981-021-00378-4

**Published:** 2021-08-16

**Authors:** Trevor A. Crowell, Haoyu Qian, Carsten Tiemann, Clara Lehmann, Christoph Boesecke, Albrecht Stoehr, Jukka Hartikainen, Stefan Esser, Markus Bickel, Christoph D. Spinner, Stephan Schneeweiß, Christiane Cordes, Norbert Brockmeyer, Heiko Jessen, Merlin L. Robb, Nelson L. Michael, Klaus Jansen, Hendrik Streeck, for the BRAHMS Study Team

**Affiliations:** 1 U.S. Military HIV Research Program, Walter Reed Army Institute of Research, Silver Spring, MD USA; 2 Henry M. Jackson Foundation for the Advancement of Military Medicine, Bethesda, MD USA; 3 MVZ Labor Krone GbR, Bad Salzuflen, Germany; 4 Uniklinik Köln, Cologne, Germany; 5 Department of Internal Medicine, Medical Faculty, University Bonn, Bonn, Germany; 6 Institut Für Interdisziplinäre Medizin, Hamburg, Germany; 7 Zentrum Für Infektiologie, Berlin, Germany; 8 Department of Dermatology and Venerology, University Hospital Essen, University of Duisburg-Essen, Essen, Germany; 9 Institute for Translational HIV Research, University Hospital Essen, University of Duisburg-Essen, Essen, Germany; 10 Infektiologikum, Frankfurt, Germany; 11 Technical University of Munich, School of Medicine, University Hospital Rechts Der Isar, Munich, Germany; 12 Praxis Hohenstaufenring, Cologne, Germany; 13 Praxis Dr. Cordes, Berlin, Germany; 14 Center for Sexual Health and Medicine, WIR-Walk In Ruhr, Bochum, Germany; 15 Interdisciplinary Immunological Outpatient Clinic, Center for Sexual Health and Medicine, Department of Dermatology, Venereology and Allergology, Ruhr Universität Bochum, Bochum, Germany; 16 Praxis Jessen2 + Kollegen, Berlin, Germany; 17 Center for Infectious Disease Research, Walter Reed Army Institute of Research, Silver Spring, MD USA; 18 Department for Infectious Disease Epidemiology, Robert Koch Institute, Berlin, Germany; 19 Institute of Virology, Medical Faculty, University Bonn, Bonn, Germany

**Keywords:** Screening practices, Sexual and gender minorities, Europe, Voluntary counseling and testing, Human immunodeficiency virus, Hepatitis C virus

## Abstract

**Background:**

HIV and hepatitis C virus (HCV) have shared routes of transmission among men who have sex with men (MSM). Routine testing facilitates early diagnosis and treatment, thereby preventing morbidity and onward transmission. We evaluated factors associated with HIV and HCV testing in a behaviorally vulnerable cohort of predominantly MSM.

**Methods:**

From June 2018 through June 2019, the BRAHMS study enrolled adults at ten German outpatient clinics that serve gender and sexual minority populations. Participants completed behavioral questionnaires that captured prior experience with HIV and HCV testing. Multivariable robust Poisson regression was used to evaluate factors potentially associated with testing in the previous 6 months.

**Results:**

Among 1017 participants with median age 33 (interquartile range 28–39) years, 1001 (98.4%) reported any lifetime history of HIV testing and 787 (77.4%) reported any HCV testing, including 16 (1.6%) known to be living with HCV. Testing within the last 6 months was reported by 921 (90.6%) and 513 (50.4%) for HIV and HCV, respectively. Recent HIV testing was more common among participants with higher education level and recent HCV testing. Recent HCV testing was more common among participants with non-cisgender identity, lifetime history of illicit drug use, hepatitis B immunity or infection, and recent HIV testing.

**Conclusion:**

Prior testing for HIV was common in this cohort, but interventions are needed to improve HCV risk stratification and access to testing. HIV testing infrastructure can be successfully leveraged to support HCV testing, but differentiated preventive care delivery is needed for some vulnerable populations.

## Introduction

For persons living with HIV unaware of their status, HIV testing is the critical first step toward diagnosis, initiation of antiretroviral therapy (ART), and further HIV care. Routine testing with prompt treatment after diagnosis drastically decreases HIV-related morbidity [1, 2], is one of the most cost-effective strategies for preventing onward HIV transmission [3, 4], and is key to curbing the HIV pandemic [5, 6].

Despite partial decreases in HIV incidence among men who have sex with men (MSM) in Germany and other Western European countries in recent years, substantial HIV transmission continues [7–11]. The European Centre for Disease Prevention and Control recommends annual testing of sexually active MSM for HIV and other sexually transmitted infections (STIs) with consideration for more frequent testing [12]. U.S. recommendations are similar [13] and add 3-monthly testing for MSM who are prescribed HIV pre-exposure prophylaxis (PrEP) [14]. Despite such recommendations, in 2016, about one-sixth of MSM living with HIV in six European countries were undiagnosed and one-third of diagnoses occurred late in the disease course, after CD4 had declined below 350 cells/μL [15]. In a survey from 13 European cities, almost two-thirds of MSM with undiagnosed HIV had evidence of infection within the past year, underscoring a need for more frequent testing to catch infection earlier in its course [16].

Due to shared routes of transmission, including sexual transmission via condomless anal intercourse, MSM who are behaviorally vulnerable to HIV are also at risk for hepatitis C virus (HCV) [17, 18]. Across Europe, an estimated 3.9 million individuals are living with chronic HCV [19] though many are unaware [20]. Outbreaks of sexually transmitted acute HCV have been documented among MSM living with HIV in Europe, the U.S., Australia, and Southeast Asia [21–26]. Coinfection with HIV and HCV worsens clinical outcomes as compared to either disease alone [27–29]. Direct-acting antiretrovirals are highly effective therapy for HCV but, as with ART for HIV, screening and diagnosis of HCV are prerequisite to therapy [30].

Data on factors associated with HCV testing are scarce, but factors associated with HIV testing have been evaluated in many settings. Studies of European MSM have identified factors associated with no or infrequent testing that included younger age, living in rural communities, lower reported number of sexual partners, less self-reported condomless anal intercourse, and low perceived HIV risk [31–37]. However, many of these prior studies were conducted before the expansive roll-out of PrEP in Germany and other European countries. In September 2017, the monthly cost of PrEP dropped about 90% to less than 50 Euros in Germany [38]. Since September 2019, PrEP has been available for only 5–10 Euros per month to behaviorally vulnerable individuals over 16 years old through statutory health insurance [39].

We evaluated factors associated with self-report of prior HIV and HCV testing upon enrollment into a cohort of predominantly MSM with sexual risk profiles suggesting vulnerability to HIV and HCV who were receiving care at ten German clinics in the era of widely available PrEP. Our objective was to determine demographic and behavioral characteristics that were associated with testing uptake in order to inform the design of targeted interventions to increase HIV and HCV testing among MSM.

## Methods

### Study population

The BRAHMS study was a prospective observational study that enrolled participants who were behaviorally vulnerable to HIV at ten German outpatient clinics and private practices that serve gender and sexual minority populations. Eligibility criteria included a non-reactive HIV test, age 18–55 years, and male sex (either at birth, chosen, or intersexual). Participants also needed, in the preceding 24 weeks, either documented diagnosis of an STI or self-reported condomless anal intercourse with 2 or more male partners known to be living with HIV or with unknown HIV status. Enrolled participants received risk reduction counseling and underwent testing for HIV and other STIs every 3 months for up to 12 months.

All participants provided written informed consent in either German or English. The study was approved by institutional review boards at all collaborating institutions.

### Data collection

Data for these cross-sectional analyses were collected at the screening and enrollment visits, which occurred 2–4 weeks apart. The screening visit included a computer-assisted self-interview (CASI) questionnaire that collected detailed information regarding participant demographics, sexual behaviors, STIs, and substance use.

To evaluate previous HIV testing experience, participants were asked, “Have you ever been tested for HIV?” If the answer was affirmative, then they were asked, “When did you last have an HIV test?” To characterize prior testing for HCV, participants were asked, “Have you ever been tested for hepatitis C?” If the answer was affirmative and the test result was negative, then they were asked, “When was the last negative test for hepatitis C?” If a participant reported a prior positive test for HCV, then they were asked “Was the hepatitis C infection treated?” with answer choices that included “yes, successfully,” “yes, without success,” “no, the infection cleared without treatment,” and “no, and I still have it.”

All participants underwent point-of-care HIV testing using the Alere HIV Combo rapid test (Abbott GmbH & Co. KG, Wiesbaden, Germany). Centralized confirmatory testing was performed using the Alinity I HIV Ag/Ab Combo assay (Abbott GmbH & Co. KG, Wiesbaden, Germany) and Aptima HIV-1 Quant Dx assay (Hologic, Marlborough, MA, USA). Hepatitis B status was determined using the Alinity HBsAg Qualitative II assay (Abbott Ireland, Sligo, Ireland), Alinity Anti-HBs assay (Abbott Ireland, Sligo, Ireland), and Alinity Anti-HBc assay (Abbott GmbH & Co. KG, Wiesbaden, Germany). HCV testing was performed using the Aptima HCV Quant Dx assay (Hologic, Marlborough, MA, USA). All testing was performed according to manufacturer instructions.

### Statistical analyses

All enrolled participants were included in these analyses. Comparisons between groups of interest were made using the Chi-squared test for categorical variables or Student’s t-test for continuous variables. In separate analyses for prior HIV testing and prior HCV testing, unadjusted and adjusted robust Poisson regression models were used to estimate risk ratios (RRs) and 95.0% confidence intervals (CIs) for pre-specified factors potentially associated with testing in the previous 6 months [40]. All enrolled participants were potentially at risk for HIV and were included in modeling of factors associated with recent HIV testing; only participants without a known diagnosis of HCV were included in modeling of factors associated with recent HCV testing since such testing would not be indicated for individuals known to be living with HCV. For each outcome, factors with p < 0.10 in unadjusted models were included in the adjusted multivariable model. All analyses were performed using Stata 15.0 (StataCorp LP, College Station, TX).

## Results

### Study population and lifetime history of testing

From June 2018 through June 2019, a total of 1017 participants enrolled with median age 33 (interquartile range 28–39) years. Participants were predominantly cisgender men (n = 1002, 98.5%), but also included five (0.5%) non-binary individuals, one (0.1%) transgender woman, one (0.1%) transgender man, one (0.1%) participant who identified as gender queer, and seven (0.7%) who did not report a gender identity at the screening or enrollment visit. Most participants self-identified as homosexual (n = 936, 92.0%). Undergraduate or higher education was reported by 535 (52.6%) and 624 (61.4%) were single or never married. HIV risk was self-reported as none/small by 438 (43.1%) participants, some by 357 (35.1%), and large/very large by 217 (21.3%) with 5 (0.5%) not responding to this question. Any lifetime history of illicit drug use was reported by 668 (65.7%) and binge drinking in the last year was reported by 345 (33.9%; Table 1).

**Table 1:** Study population characteristics, overall and by testing history

	Overall (n = 1017)	Recent HIV testing			Recent HCV testing		
		No HIV test for > 6 months (n = 96)	Tested for HIV < 6 months ago (n = 921)	p	No HCV test for > 6 months (n = 488)	Tested for HCV < 6 months ago (n = 513)	p
Age (years)				**0.008**			0.16
< 25	96 (9.4%)	16 (16.7%)	80 (8.7%)		51 (10.5%)	45 (8.8%)	
25–35	496 (48.8%)	51 (53.1%)	445 (48.3%)		223 (45.7%)	265 (51.7%)	
> 35	425 (41.8%)	29 (30.2%)	396 (43.0%)		214 (43.9%)	203 (39.6%)	
Gender identity				0.075			0.17
Cisgender man	1002 (98.5%)	93 (96.9%)	909 (98.7%)		484 (99.2%)	503 (98.1%)	
Transgender man	1 (0.1%)	0 (0.0%)	1 (0.1%)		0 (0.0%)	1 (0.2%)	
Transgender woman	1 (0.1%)	0 (0.0%)	1 (0.1%)		1 (0.2%)	0 (0.0%)	
Non-binary	5 (0.5%)	0 (0.0%)	5 (0.5%)		0 (0.0%)	5 (1.0%)	
Gender queer	1 (0.1%)	0 (0.0%)	1 (0.1%)		0 (0.0%)	1 (0.2%)	
Missing/unknown	7 (0.7%)	3 (0.3%)	4 (0.4%)		3 (0.6%)	3 (0.6%)	
Sexual orientation				**< 0.001**			**0.037**
Homosexual	936 (92.0%)	86 (89.6%)	850 (92.3%)		447 (91.6%)	475 (92.6%)	
Bisexual	48 (4.7%)	6 (6.3%)	42 (4.6%)		28 (5.7%)	19 (3.7%)	
Heterosexual	1 (0.1%)	1 (1.0%)	0 (0.0%)		1 (0.2%)	0 (0.0%)	
Other or no label	23 (2.3)	0 (0.0%)	23 (2.5%)		6 (1.2%)	17 (3.3%)	
Missing/unknown	9 (0.9%)	3 (3.1%)	6 (0.7%)		6 (1.2%)	2 (0.4%)	
Education level				**< 0.001**			0.60
Less than secondary	199 (19.6%)	25 (26.0%)	174 (18.9%)		102 (20.9%)	94 (18.3%)	
Secondary school	283 (27.8%)	40 (41.7%)	243 (26.4%)		137 (28.1%)	143 (27.9%)	
Undergraduate	171 (16.8%)	11 (11.5%)	160 (17.4%)		85 (17.4%)	85 (16.6%)	
Master’s or doctorate	364 (35.8%)	20 (20.8%)	344 (37.4%)		164 (33.6%)	191 (37.2%)	
Marital status				0.71			**0.024**
Single/never married	624 (61.4%)	63 (65.6%)	561 (60.9%)		282 (57.8%)	334 (65.1%)	
Married	120 (11.8%)	8 (8.3%)	112 (12.2%)		57 (11.7%)	60 (11.7%)	
Cohabitating	195 (19.2%)	17 (17.3%)	178 (19.3%)		104 (21.3%)	86 (16.8%)	
Separated/widowed	73 (7.2%)	7 (7.3%)	66 (7.2%)		40 (8.2%)	33 (6.4%)	
Other/unknown	5 (0.5%)	1 (1.0%)	4 (0.4%)		5 (1.0%)	0 (0.0%)	
Self-perceived HIV risk^a^				**0.001**			0.32
None/small	438 (43.1%)	36 (37.5%)	402 (43.6%)		197 (40.4%)	235 (45.8%)	
Some	357 (35.1%)	37 (38.5%)	320 (34.7%)		184 (37.7%)	170 (33.1%)	
Large/very large	217 (21.3%)	20 (20.8%)	197 (21.4%)		104 (21.3%)	106 (20.7%)	
Missing/unknown	5 (0.5%)	3 (3.1%)	2 (0.2%)		3 (0.6%)	2 (0.4%)	
Illicit drug use in lifetime				**0.009**			** < 0.001**
No	327 (32.2%)	34 (35.4%)	293 (31.8%)		171 (35.0%)	155 (30.2%)	
Yes	668 (65.7%)	56 (58.3%)	612 (66.4%)		297 (60.9%)	356 (69.4%)	
Missing/unknown	22 (2.2%)	6 (6.3%)	16 (1.7%)		20 (4.1%)	2 (0.4%)	
Binge drinking in last year^b^				0.074			0.71
No	608 (59.8%)	57 (59.4%)	551 (59.8%)		291 (59.6%)	309 (60.2%)	
Yes	345 (33.9%)	28 (29.2%)	317 (34.4%)		169 (34.6%)	169 (32.9%)	
Missing/unknown	64 (6.3%)	11 (11.5%)	53 (5.8%)		28 (5.7%)	35 (6.8%)	
Hepatitis B status				0.051			**0.005**
Susceptible	149 (14.7%)	22 (22.9%)	127 (13.8%)		89 (18.2%)	59 (11.5%)	
Immune	866 (85.2%)	74 (77.1%)	792 (86.0%)		399 (81.8%)	452 (88.1%)	
Infected	2 (0.2%)	0 (0.0%)	2 (0.2%)		0 (0.0%)	2 (0.4%)	

Participant characteristics were assessed at study screening and enrollment visits. All data are presented as n (column percentage). p-values were calculated using Pearson’s Chi-squared test and significant results (p < 0.05) are shown in bold. Sixteen participants known to be living with HIV were not included in the evaluation of recent HCV testing, since HCV diagnostic testing would not routinely be indicated for such individuals

^a^Self-perceived HIV risk was assessed with the question, “Thinking about the sex you had in the past 12 months, to what extent would you consider yourself at risk of getting HIV?” with answers provided via a 5-point Liekert scale from “no risk” to “very large risk.”

^b^Binge drinking was defined as having six or more drinks during one occasion once or more per month during the past year

^c^Hepatitis B status was categorized as “susceptible” if all surface antigen, surface antibody, and core antibody were all non-reactive; “immune” if only the surface antibody or core antibody was reactive; and “infected” if the surface antigen was detectable

**Fig. 1:**
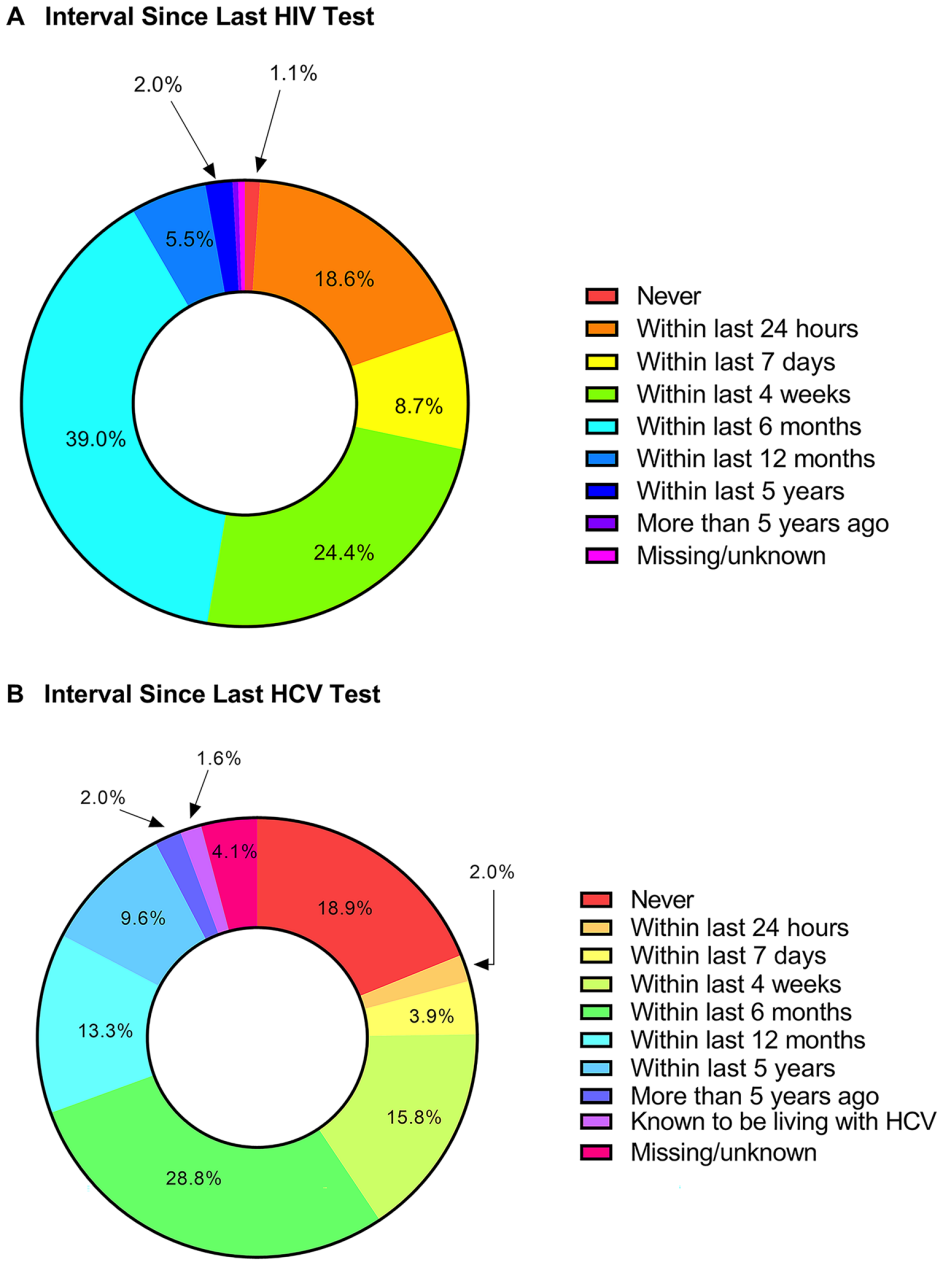
Interval since last reported HIV and HCV tests. At cohort enrollment, participants were asked if they had ever been tested for HIV and hepatitis C. If the answer was affirmative, they were asked when the last test was performed. Participants who were known to be living with hepatitis C were categorized separately because repeat testing is not indicated in this population

Of the 1017 participants enrolled, 1001 (98.4%) reported having been tested for HIV at least once in their lifetime, including 921 (90.6%) who reported HIV testing within the 6 months prior to enrollment (Fig. 1A).

Any lifetime history of testing for HCV was reported by 787 (77.4%) participants, including 16 (1.6%) known to be living with HCV and 513 (50.4%) who reported HCV testing within the 6 months prior to enrollment (Fig. 1B).

### Factors associated with recent HIV and HCV testing

After adjusting for potentially confounding factors, HIV testing within the 6 months prior to enrollment was more common among participants with advanced education and recent HCV testing (Table 2). Recent HCV testing was more common among participants with non-cisgender identity, lifetime history of illicit drug use, hepatitis B immunity or infection, and recent HIV testing (Table 2).

**Table 2:** Factors associated with testing for HIV and hepatitis C virus in the last 6 months

	Recent HIV testing			Recent HCV testing		
	n tested/N at risk	Unadjusted risk ratio (95% CI)	Adjusted risk ratio (95% CI)	n tested/N at risk^f^	Unadjusted risk ratio (95% CI)	Adjusted risk ratio (95% CI)
Age (years)						
< 25	80/96	Reference	Reference	45/96	Reference	
25–35	445/496	1.08 (0.98–1.18)	1.03 (0.94–1.12)	265/488	1.16 (0.92–1.46)	
> 35	396/425	**1.12 (1.02–1.23)**	1.08 (0.99–1.18)	203/417	1.04 (0.82–1.31)	
Gender identity						
Cisgender man	909/1002	Reference		503/987	Reference	Reference
Transgender/other/unknown	12/15	0.88 (0.68–1.14)		10/14	**1.40 (1.00–1.96)**	**1.52 (1.23–1.87)**
Sexual orientation						
Homosexual	850/936	Reference		475/922	Reference	
Bisexual	42/48	0.96 (0.86–1.07)		19/47	0.78 (0.55–1.12)	
Heterosexual/other/unknown	29/33	0.97 (0.85–1.10)		19/32	1.15 (0.86–1.55)	
Education level						
Less than secondary	174/199	Reference	Reference	94/196	Reference	
Secondary school	243/283	0.98 (0.91–1.05)	0.99 (0.92–1.05)	143/280	1.06 (0.88–1.28)	
Undergraduate	160/171	**1.07 (1.00–1.14)**	**1.08 (1.01–1.15)**	85/170	1.04 (0.84–1.29)	
Master’s or doctorate	344/364	**1.08 (1.02–1.15)**	**1.06 (1.00–1.12)**	191/355	1.12 (0.94–1.34)	
Marital status						
Single/never married	561/624	Reference		334/616	Reference	Reference
Married/cohabitating	290/315	1.03 (0.98–1.07)		146/307	0.88 (0.77–1.02)	0.88 (0.77–1.00)
Separated/widowed	66/73	1.00 (0.93–1.09)		33/73	0.84 (0.65–1.09)	0.82 (0.63–1.08)
Other/unknown	4/5	0.89 (0.57–1.38)		0/5	–^e^	–^e^
Self-perceived HIV risk^a^						
None/small/unknown	404/443	Reference		237/437	Reference	Reference
Some	320/357	0.98 (0.94–1.03)		170/354	0.88 (0.77–1.02)	0.89 (0.78–1.01)
Large/very large	197/217	1.00 (0.95–1.05)		106/210	0.93 (0.79–1.09)	0.92 (0.80–1.07)
Illicit drug use in lifetime						
No^b^	309/349	Reference		157/348	Reference	Reference
Yes	612/668	1.03 (0.99–1.08)		356/653	**1.21 (1.06–1.38)**	**1.15 (1.01–1.31)**
Binge drinking in last year^c^						
No	551/608	Reference		309/600	Reference	
Yes	317/345	1.02 (0.98–1.06)		169/338	0.96 (0.84–1.10)	
Missing/unknown	53/64	0.91 (0.81–1.02)		35/63	1.08 (0.85–1.36)	
Hepatitis B status^d^						
Susceptible	127/149	Reference	Reference	59/148	Reference	Reference
Immune	792/866	**1.07 (1.00–1.15)**	1.04 (0.97–1.11)	452/851	**1.33 (1.08–1.64)**	**1.24 (1.01–1.50)**
Infected	2/2	**1.17 (1.10–1.25)**	1.04 (0.94–1.16)	2/2	**2.51 (2.06–3.06)**	**2.54 (1.78–3.61)**
Tested for HIV within 6 months						
No		–		5/95	Reference	Reference
Yes		–		508/906	**10.6 (4.53–25.1)**	**10.4 (4.44–24.6)**
Tested for HCV within 6 months						
No	398/488	Reference	Reference		–	
Yes	523/529	**1.21 (1.16–1.27)**	**1.21 (1.16–1.26)**		–	

Multivariable Poisson regression with robust error variance was used to estimate risk ratios and 95% confidence intervals (CIs) for factors potentially associated with prior testing for HIV and hepatitis C in the 6 months prior to study entry. For each outcome, factors with p < 0.10 in unadjusted models were included in the adjusted model. Statistically significant (p < 0.05) risk ratios are shown in bold

^a^Self-perceived HIV risk was assessed with the question, “Thinking about the sex you had in the past 12 months, to what extent would you consider yourself at risk of getting HIV?” with answers provided via a 5-point Liekert scale from “no risk” to “very large risk.”

^b^To enable model convergence, missing data were included in the reference category

^c^Binge drinking was defined as having six or more drinks during one occasion once or more per month during the past year

^d^Hepatitis B status was categorized as “susceptible” if all surface antigen, surface antibody, and core antibody were all non-reactive; “immune” if only the surface antibody or core antibody was reactive; and “infected” if the surface antigen was detectable

^e^No participants with “other/unknown” marital status reported HCV testing in the last 6 months; to enable model convergence, these participants were collapsed into the “single/never married” reference category

^f^Only participants not previously known to be living with HIV were included in the evaluation of recent HCV testing, since HCV diagnostic testing would not routinely be indicated for people already known to be living with HIV

## Discussion

Prior screening for HIV and HCV was common in this cohort of predominantly cisgender men with known risk factors for infection who were receiving care at specialized clinics. Remarkably, over half of participants had been screened for HIV within the month prior to enrollment, perhaps reflecting some sampling bias due to the targeted enrollment of participants with sexual risk behaviors for which current guidelines recommend frequent STI testing. Fewer than 5% of participants had not been screened for HIV within the year prior to enrollment in our study, while a less targeted online survey of German MSM found that 35% of respondents had never been tested for HIV and 62% had not been tested within the preceding 12 months [31]. Our study was conducted at German outpatient clinics and private practices that serve sexual and gender minorities and are likely to be particularly attuned to HIV risk assessment and screening needs of these key populations.

Non-cisgender identity was associated with significantly increased HCV testing uptake in our study, but no significant difference in HIV testing. Studies in other settings have found that HIV testing tends to be similar [41] or lower [42–44] among transgender, non-binary, and other non-cisgender people as compared to other key populations. Our finding of increased HCV testing in non-cisgender participants may reflect provider biases toward testing in this population, though inferences should be made with caution due to the small sample size of persons identifying as non-cisgender in our study and the small overall effect size associated with this factor.

There was a strong correlation between recent testing for HIV and HCV, which is appropriate given the potential for shared routes of transmission and guideline recommendations for HCV testing to follow HIV testing due to sexual risk [12]. The World Health Organization has set a target to diagnose 90% of people living with HCV by 2030 [45], but only about 20% of the estimated 71 million people worldwide who are living with chronic HCV had been diagnosed by 2015 [46]. Despite shared risk factors for acquisition, prior studies have shown that HCV testing tends to be less common than HIV testing among vulnerable populations [47, 48]. The testing gap appears to be smaller when HIV and HCV testing are offered together as one package [49, 50]. Leveraging existing screening infrastructure focused on HIV diagnosis could be helpful for facilitating earlier HCV diagnosis in some populations, but even in our study only about three-quarters of participants had ever been tested for HCV and about half of those at risk had been tested in the previous 6 months. Further efforts are need to ensure adequate linkage to care for people at risk for HCV, raise awareness, and scale-up testing services to include point-of-care options [51].

Nearly half of study participants perceived themselves to be at low or no risk for HIV despite entry criteria that required risk behaviors. Self-perception of HIV risk was not associated with increased recent HIV or HCV testing uptake. One meta-analysis found that HIV risk perception was associated with increased uptake of HIV testing across studies mostly conducted in Africa and the Americas [52]. Among the few European studies on this topic, one conducted in African migrant communities in London observed a significant association between HIV risk perception and testing uptake among women but not men [53], while one in Paris found an association among men but not women [54]. A German study observed a correlation between perceived risk and HIV testing in MSM [35]. Conflicting data on this topic underscore the variability of risk perception across communities, the ambiguity of measuring risk perception across studies, and likely multifactorial motivations for testing. Our findings suggest that, among German MSM, there is a need for outreach and extension of prevention interventions outside the clinical setting to encourage testing of individuals who do not self-identify their risk.

Most of the data in these analyses were assessed by self-report and could be susceptible to social desirability bias, though collection of data by CASI would be expected to reduce this bias. Data were collected cross-sectionally at enrollment into a study that included routine HIV and HCV screening, so analyses were based primarily on historic experiences and temporal associations could not be evaluated. The study was conducted at German outpatient clinics and private practices serving sexual and gender minorities; our findings may not be generalizable to other populations or settings.

## Conclusion

Despite high overall uptake of HIV and HCV testing among at-risk German MSM, there is room for improvement particularly in uptake of HCV testing. Lessons learned from the successful deployment of frequent HIV screening to vulnerable sexual and gender minority populations in Germany can be applied to testing for HCV and could inform differentiated preventive care delivery to other populations.

## Data Availability

The Henry M. Jackson Foundation for the Advancement of Military Medicine (HJF) and the Water Reed Army Institute of Research (WRAIR) are committed to safeguarding the privacy of research participants. Distribution of data will require compliance with all applicable regulatory and ethical processes, including establishment and approval of an appropriate data-sharing agreement. To request a minimal data set, please contact the data coordinating and analysis center (DCAC) at PubRequest@hivresearch.org and indicate the BRAHMS/RV500 study along with the name of the manuscript.

## Abbreviations

ART: Antiretroviral therapy
CASI: Computer-assisted self-interview
CI: Confidence intervals
HCV: Hepatitis C virus
HIV: Human immunodeficiency virus
MSM: Men who have sex with men
PrEP: Pre-exposure prophylaxis
RR: Risk ratio
STIs: Sexually transmitted infections
